# Budgeting for PACS

**DOI:** 10.2349/biij.4.4.e32

**Published:** 2008-10-01

**Authors:** LH Sim

**Affiliations:** PACS Support Unit, Department of Radiology, Princess Alexandra Hospital, Brisbane, Australia

**Keywords:** PACS, budget, radiology, purchase

## Abstract

There are a number of models for the acquisition of digital image management systems. The specific details for development of a budget for a PACS/RIS acquisition will depend upon the acquisition model – although there are similarities in the overarching principles and general information, particularly concerning the radiology service requirements that will drive budget considerations.

While budgeting for PACS/RIS should follow the same principles as budgeting for any new technology, it is important to understand how far the implementation of digital image management systems can reach in a healthcare setting. Accurate identification of those elements of the healthcare service that will be affected by a PACS/RIS implementation is a critical component of successful budget formation and of the success of any business case and subsequent project that relies on those budget estimates.

A budget for a PACS/RIS capital acquisition project should contain capital and recurrent elements. The capital is associated with the acquisition of the system in a purchase model and capital budget may also be required for upgrade – depending upon a facility’s financial management processes.

The recurrent (or operational) cost component for the PACS/RIS is associated with maintaining the system(s) in a sustainable operational state.

It is also important to consider the service efficiencies, cost savings and service quality improvements that PACS/RIS can generate and include these factors into the economic analysis of any proposal for a PACS/RIS project.

## INTRODUCTION

Moving a radiology service from a film-based service to a digital image management (filmless) service through implementation of a Picture Archive Communication System (PACS) and associated Information Systems (e.g. Radiology Information System (RIS)) requires consideration of a wide range of relevant topics [1, 2], including:

- Medical imaging service requirements;
- User requirements;
- Workflow analysis;
- State of the technology (i.e. current systems capability);
- State of the market (i.e. current product offerings);
- Indicative financial expenditures – capital and recurrent budgets; and,
- Cost/benefit or cost/effectiveness analysis.

This information is generally part of the investigation as to whether a PACS project is a feasible initiative. It should be a preliminary phase that informs a decision to commence work on technical and performance specifications [3] prior to the establishment of a procurement exercise.

The budgeting component is a very important part of the business case development [4] and is crucial to any assessment of the economic viability [5] of the proposed PACS/RIS initiative.

## BUDGETING PRINCIPLES

Budgeting for PACS/RIS should follow the same principles as budgeting for any new technology. However, implementation of digital image management systems will have far-reaching effects in a healthcare setting – impacting clinical workflows and creating opportunities for improved efficiencies and quality improvements. Accurate identification of the various elements of the healthcare service that will be affected by a PACS/RIS implementation is a critical component of the budget formation process and of any business case and subsequent project implementation that relies on those budget estimates.

Budgets are usually identified as capital and recurrent [6]. It is no different for PACS/RIS. The capital budget estimate identifies the probable costs to purchase and implement the technology. The recurrent budget estimate represents the projected future costs of managing and maintaining the system in a sustainable operational state.

## PACS ACQUISITION FINANCIAL MODELS

When acquiring a PACS/RIS, the specific nature of the budget will depend upon the model of acquisition. There are a number of such models, including:

1. Traditional purchase: In this model the technology is purchased outright and owned by the institution. It is usually managed by the institution (i.e. PACS Administration) with the vendor providing technical support under a service contract arrangement. It is possible (but not yet common) for support to be provided by third-party providers.
2. Application Service Provider (ASP) Model: In this model, the facility purchases a “service” from the vendor. The vendor then implements and manages the system with charges based on fee-per-service arrangements. The facility does not own the hardware or software (but should own the information). This model moves some of the capital acquisition costs into the recurrent budget, spreading that expenditure across the life of the system.
3. Hybrid ASP Models: The extent of the ASP model may be limited to (e.g.) archiving with the facility taking responsibility for and ownership of (e.g.) reporting workstations and interface hardware/software.
4. Leasing Models: Rather than purchasing the technology outright, a facility may choose to lease. This effectively moves all of the capital budget requirement into the recurrent budget. By doing so, it spreads the capital expenditure across the life of the system. Leasing can also have some financial incentives (e.g. taxation benefits) in a private sector context.

In the public sector, the most common method of acquiring this technology has been through a traditional purchase (i.e. Model (a)) where the facility buys and owns the system.

## ANALYSIS OF BUDGET COMPONENTS FOR TRADITIONAL PURCHASE OF PACS/RIS

The following discussion is based on a public sector PACS/RIS traditional purchase acquisition model. It illustrates how a capital and recurrent budget might be established for this model. The discussion presents questions that will need to be asked and answered in order to provide information necessary to establish project scope and obtain accurate budget estimates. Similar questions will also be pertinent to establishing budgets for the other acquisition models noted above.

### Capital Budget Component Items for establishment of a PACS/RIS Acquisition Project:

#### Image Archive

The image archive in a PACS is the repository of medical images and must be able to store images and allow retrieval of images for clinical use. A PACS archive will typically consist of a number of levels of storage in order to balance cost, reliability and speed of retrieval.

Level 1 storage is designed to retain images in a high availability state for rapid retrieval for use in patient diagnosis and therapy. This is called the period of clinical usefulness and may range from hours to years depending upon the patient condition. Level 1 storage is sometimes referred to as archive cache and usually consists of high quality, high reliability hard disk arrays. Images outside the defined period of clinical usefulness are usually stored on more cost-effective storage media, to meet mandated legislative storage requirements — e.g. digital versatile disk (DVD), compact disk (CD), magnetic tape and more recently on lower cost high volume disk arrays. This is Level 2 storage and comprises the major volume of the image archive.

The budget requirement for the archive will depend upon the storage size requirements so the following questions need to be addressed:

- How much storage is required initially?
- What is the projected growth in storage requirements?
- What is the defined period of clinical usefulness and how much cache is needed?
- What is the disaster recovery strategy?

The answers to these questions will depend upon factors such as:

1. The clinical workload of the facility and projected growth in that workload: The PACSNet unit in the Centre for Evidence Based Purchasing of the National Health Service (NHS) in England has produced a useful storage calculator tool for estimating PACS storage requirements. This calculator can be found at the PACSNet website [7]. One strategy for storage acquisition may be to purchase a minimum amount of initial storage and factor in future budget allocation to add to the archive storage as growth in requirements demands, thereby achieving the benefits of future decreases in storage costs.
2. Introduction of new and additional imaging technologies (e.g. Multi Detector Computed Tomography (MDCT)) that may generate additional data growth.
3. The records retention and archiving legislation and policy for the relevant jurisdiction or facility will determine the minimum records retention time and hence is an important factor in the establishment of archive storage size. The archive will need to be sized to store images for at least the period of time mandated by legislation and/or policy. There is a useful discussion of record retention practices in the USA in the article by Rinehart-Thompson [8].
4. Image cache should be adequately sized to store a majority of relevant prior studies for rapid retrieval by radiologists when reporting current imaging studies. This will require a definition (by clinicians) of the period of clinical usefulness to allow cache storage volumes to be calculated.
5. Disaster recovery involves mitigation of risks of data loss due to events such as fire and natural disasters by having a copy of the archive located in a separate location to the primary archive. This requirement may dictate a need for a third level of archive such as a lower cost tape archive located off-site. In some cases disaster recovery requirements might involve full replication of the archive in a duplicate data centre.

#### Workstations

Medical imaging studies are (usually) viewed and reported by a radiologist and a report on the findings is produced. This process is called primary diagnosis. When the radiologist’s report and images are viewed by the patients’ treating doctor, it is called clinical review.

There are two main types of PACS workstations — diagnostic workstations for primary diagnosis and clinical review stations for clinical review. Because of the higher performance requirements, a diagnostic workstation is usually considerably more expensive than a clinical review workstation.

Budget requirements for workstations will depend upon the answers to questions such as:

- How many diagnostic workstations are required? Should diagnostic workstations be deployed outside of radiology, where primary care decisions are made? (e.g. Emergency Department, Intensive Care Unit)
- Are clinical review workstations within the scope of the project budget or are these to be funded from the user areas? What monitor specifications are required for primary diagnosis and for clinical review? (e.g. colour/monochrome, spatial resolution, luminance, etc) [9]

#### Server hardware

The types, numbers and specifications of server hardware components will need to be scoped to deal with the projected workload and required levels of redundancy and resilience within the system. PACS has become mission critical in the filmless environment so it is important that adequate redundancy exists to support radiology business continuity in the event of hardware failure [10,11]. This scoping will require input from information technology specialists and will require answers to question such as:

- What level of database, archive, RIS, web server and other miscellaneous server power is required?
- Is there a requirement for redundant power supply in all servers?
- Is server hardware required to create “test” system environments?
- Should there be discrete duplicate (backup) servers with automatic failover or should there be a complete duplicate data centre?

#### Image Distribution

The strategy for image and results distribution within the hospital will need to be defined. A common strategy is to use a web-based image distribution system. In this circumstance it is necessary to determine if the existing hospital Personal Computers (PCs) are adequate for ward and clinic viewing of images or if there is a need for clinical viewing stations with higher specification monitors to be incorporated into the project budget.

The digitisation of medical images facilitates a technical image management base that makes it possible for:

- hospital clinicians to view images remotely;
- referring doctors to receive images and reports electronically;
- radiologists to report remotely (e.g. from home);
- public sector imaging studies to be forwarded for reading to contracted private sector radiologists for reading; and
- hospitals to access teleradiology [12] providers to obtain radiology reporting services.

These initiatives will generate questions about:

- the funding models for provision of ward and clinic monitors;
- remote access services requirements;
- bandwidth requirements;
- remote radiologist workstations;
- security services; and
- teleradiology arrangements.

A number of these issues are complex – involving more than just the technical implementation of a PACS/RIS. Decisions and policies will be required as to the scope of provision of these facilities and arrangements within the PACS/RIS project versus separately scoped service provision projects for (e.g.) teleradiology and “user pays” models of funding for (e.g.) provision of remote access arrangements and review station hardware.

#### Modality Interfaces

The interface of modalities to the PACS should be a relatively straightforward set of tasks in a DICOM [13] conformant environment. However it should not be assumed that modality connections will be achieved without difficulties. Allowance needs to be made for these tasks and should include:

- PACS vendor input; and
- Liaison with and input from modality vendors.

Consideration is required of the number of modalities to be linked and whether there is any licensing or implementation cost attached to each modality connection.

#### Licensing

The licensing model will also have a bearing on cost. Questions such as …

- Is the licensing model perpetual or recurrent?
- Is the licensing model based on a “total seats” model or a concurrent users’ model?

… will impact upon both capital and recurrent budgets for PACS, web-based distribution applications and RIS. Consequently it is important to gather information on the total number of users and the likely maximum number of concurrent users for each of these applications.

#### Image Viewing for Specialised Purposes

Diagnostic and clinical review workstations will satisfy the majority of requirements for radiology reporting and image review in wards and clinics. However there are other “specialty” viewing areas that will require separate attention. These include operating theatres and clinical meetings. The following questions should arise:

- How will images be viewed in operating theatres?
- How will images be viewed in clinical meeting rooms?
- What numbers and types of projection facilities will be required?

#### Network Infrastructure

The required bandwidth for image distribution within the radiology department and across the hospital can be estimated from existing image distribution data. It may be necessary to undertake a manual workflow analysis, counting the number of studies, films and images distributed in the current environment in order to obtain that data. A budget allowance for these tasks should be included.

Specialist network infrastructure advice will be required to determine if the existing network provides adequate bandwidth. Allowance for provision of such advice (either internally from the Information Technology (IT) department or via external consultancy) should also be made. Depending upon the state and capacity of the existing network, a budget allowance for network enhancements may be required.

#### Interfaces

It should not be assumed that interfacing the PACS, RIS and Hospital Information System (HIS) will be without cost. In cases where separate PACS and RIS vendors are involved, this will almost certainly not be the case. Similar considerations apply for interface from the RIS to the HIS and Electronic Medical Record (EMR) if one exists.

The following information will be required in order to frame this budget component:

- PACS/RIS Interface: Are the integration interfaces and license costs included?
- RIS/HIS/EMR Interface: What type of RIS interfaces to other information systems are required? Is there an existing EMR and is an interface to this record part of the project requirement? What are the interface implementation costs?

#### Data Migration

It is also necessary to consider how legacy imaging information is to be managed and include the costs associated with this in the budget:

- If the PACS/RIS acquisition is for a radiology service that is currently film-based, decisions will need to be made concerning the level of existing (film-based) image archive required to be transferred to digital form. In most circumstances this involves manual scanning of studies into the digital archive and movement of associated reports into the RIS. This body of work must be scoped and costed into the project budget.
- If the PACS/RIS acquisition is to replace an existing digital image management system then data migration from the legacy PACS and RIS must be considered and similarly scoped and costed for the project budget.

Decisions will be required as to whether legacy data (e.g. reports from the exiting RIS, images from the existing PACS archive) are to be migrated or not. If migration is to occur, a decision will be required on how much data will be retained. This decision will be influenced by record retention requirements and perceived clinical needs. Estimates of the likely costs of the data migration work will be required. A further consideration is the time required for migration of legacy image archives. This time can be significant and although this is not a direct economic factor, it can have a significant effect upon project timelines. Therefore accurate knowledge of the likely migration times is an important factor in reaching a decision about data migration and in terms of overall project cost.

#### PACS/RIS System Accommodation

There will be a requirement for physical space to house the PACS/RIS hardware and also to house the PACS Support staff. This space may need to be fitted out as a computer data centre with associated infrastructure (e.g. raised floor, cooling, fire suppression, uninterruptible power supply, etc.).

It will be necessary to determine if there is an available computer room or data centre where this hardware can be located, and if so what is the cost of that location. Alternatively it will be necessary to establish a dedicated PACS data centre with its associated establishment and running costs.

It will also be necessary to determine the availability and cost of office accommodation for the PACS Support staff.

#### Optional System Tools

A number of vendors provide optional system tools to assist with fault monitoring and performance surveillance. The requirements for and costs of these optional tools need to be evaluated, decisions for inclusion reached and budget allowance made.

Examples of such options may include:

- Automated system backup tools
- Monitor performance dashboards
- Server and database fault monitors
- Disk array monitors.

It should not be assumed that automated systems such as these (that can greatly assist in supporting these systems) are included in the purchase price.

#### Change Management

As the digital environment introduces new and potentially different workflows into the radiology department and the hospital generally, there will be costs associated with these changes that need to be factored into the budget.

There will be costs associated with training radiology staff and other system users. There is also a significant requirement for project planning and project monitoring. This invariably requires the release of hospital resources to attend meetings. It may be necessary to factor the costs of these resources into the project budget so that effective release of the required resources can be obtained.

The issues of change management associated with PACS implementation are well recognised [14-17]. It may be appropriate to establish a change management program (either internally or through the use of external consultancies) to manage the significant work process changes associated with a PACS/RIS implementation.

#### Additional Considerations

As well as allowing for these components, the PACS/RIS project will need to look at the state of its existing imaging equipment:

- If the facility uses conventional film-based radiography, an upgrade of plain film modalities will be required to computed radiography (CR) or digital radiography (DR).
- If the facility uses analogue fluoroscopy, an upgrade may be required to digital fluoroscopy or secondary capture devices will be required for image digitisation.
- If the facility uses analogue ultrasound, an upgrade will be required to digital ultrasound, or secondary capture devices will be required for image digitisation.
- Digital imaging modalities (e.g. CT, Magnetic Resonance Imaging (MRI)) will need to be assessed to see if they require additional components (e.g. DICOM Modality Worklist) in order to achieve maximum efficiency from the PACS/RIS system.
- It may be necessary to undertake a review of, and possible modifications to, reading room design to optimise the digital reading room environment [18, 19]

### Recurrent Budget Components (PACS/RIS)

#### System Service Contract

This is much more than a simple hardware “break/fix” arrangement. It will involve hardware elements, software support, emergency response arrangements, hours of coverage, may include upgrades and will be complicated by the component warranty arrangements.

A PACS/RIS service contract may be an annual fee that is somewhere in the vicinity of 10% to 20% of the capital cost of the system. Consequently it is important to obtain accurate estimates of likely service contract costs for various levels of support to inform budget decisions.

In establishing the service contract budget it is important to look very carefully at the component warranties and ensure that these are factored in. Some components may have a three year warranty. Others may have a warranty that is as short as three months. Software support costs may not include a warranty period.

Generally, the hardware service costs will rise to a plateau over the first three years of the system’s life cycle, as the various warranties are exhausted. Also there may be price increases factored in for general inflation. These arrangements are often included in service contracts and allow the vendor to increase the service costs in accordance with an agreed formula developed during the negotiation of the various contracts. It may not be known what the inflation factor is in a budget framing exercise – until the contract is finalised – but allowance does need to be made for inflationary factors in the out years of a service contract.

As well as the hardware costs, the service contract should include software and application support. PACS and RIS use complex database structures and are often based on proprietary server platforms. These systems require specialised software engineering support. This is often delivered remotely through a 24/7 support centre. Support costs generally reflect the support requirements. They are a major part of the recurrent budget and they are a critical consideration during contract negotiations. These support costs will depend upon the model applied and answers to question such as:

- Is a 24/7 coverage required, with proactive database monitoring?
- What level of response to logged calls is required? (30 min, 2 hours, next day?)
- What access to on-site field service engineers is required?
- What is the required response time for a field service engineer?

#### Local PACS/RIS Administration and Support

With a large PACS implementation, there is a requirement for a local, facility-owned support unit to manage the application and the vendor. A PACS Administration unit will provide services that are not usually provided through the vendor support models and include the following:

- On-site PACS Administration – day-to-day system management, upgrade planning, vendor support management.
- User training and troubleshooting
- System backups
- Higher level (computer-based) clerical activities that may require higher salaries.
- Additional internal IT support (e.g. network support).

#### System Upgrades

Upon completion of an initial PACS/RIS implementation project, there is a need to recognise that the technology will depreciate (both in value and relative functionality) and there will be a requirement to upgrade at some point in the future. It is not unusual for a PACS/RIS to need at least one version upgrade per year. The licensing for upgrades is often included within the purchase (or service) contracts. However, the facility will most likely need to pay the vendor for the professional services to implement the upgrade.

System upgrades also require inputs from the PACS Support unit – for planning meetings, training and for on-site supervision of the various component implementations. Often, upgrades are performed outside normal working hours and on weekends – to minimise disruption to the radiology department - this requires additional (local) budgeting for:

- staff absences at training courses; and,
- staff overtime payments.

Vendor’s charges for the inputs to upgrade projects can include the project planning, project management, engineering inputs (from the vendor and all subcontractors), deliveries and on-site implementation services, as well as the hardware and software components.

## SAVINGS AND QUALITY IMPROVEMENTS – THE OTHER HALF OF THE EQUATION

Calculating the purchase, upgrade and running costs of a PACS/RIS is really only half of the budgeting task. On the other side of the equation are the cost savings, efficiencies and service improvements that this technology can bring to an imaging facility.

Savings can include:

- Film costs
- Film stationery costs (packets, jackets, envelopes, etc)
- Chemistry costs
- Film storage and handling costs (including space and file room staff)
- Processor purchase and running costs
- Transcription costs (if Voice Recognition is included in the PACS/RIS)

The incidence of lost studies is vastly reduced with digital image management, so the need for repeat studies is also reduced. There are various claims that radiographers can work more productively in a digital environment. This can support faster patient throughput, involvement in value-adding clinical image management (e.g. 3-D reconstruction) or a combination of improved productivity and increased value add to the imaging process [20-22].

The immediate availability of images to the referring clinicians in a PACS environment is a direct quality improvement for patient treatment considerations and can lead to shorter waiting times to diagnosis and treatment and shorter length of stay for admitted patients [23].

In addition, the implementation of a PACS/RIS can lead to increased radiologist efficiency and more effective capture of actual examination data and patient throughput information. The RIS can facilitate more effective billing. These factors have the potential to improve revenues as well as the quality of patient services [24, 25].

## CONCLUSION

Budgeting for a PACS/RIS is not a simple process – but the general principles of budgeting apply. Budget estimates to support consideration of a PACS/RIS project must be as accurate as possible with all the elements that contribute to costs considered. Direct cost savings, workflow efficiencies and service quality improvements must also be considered. These include:

### Capital Costs

- Capital purchase costs of the PACS/RIS.
- Installation and commissioning costs
- Any costs associated with imaging equipment upgrades
- Infrastructure costs (e.g. datacentre, network, PCs for image distribution)
- Change management

### Recurrent Costs

- Staff and accommodation
- Consumables
- Ongoing training
- Upgrade costs.

It is also important to consider the service efficiencies, cost-savings and service quality improvements that PACS/RIS can generate (noted above). These factors should be part of any economic justification or business case analysis. The results of that analysis can then inform a cost/benefit or cost/justification assessment as part of the budget approval processes associated with major PACS/RIS projects.
